# Supplementary material for: The Evolution and Characterization of the RNA Interference Pathways in Lophotrochozoa
Source: Genome Biol Evol. 2024 May 7;16(5):evae098. doi: 10.1093/gbe/evae098 (PMC11114477; doi:10.1093/gbe/evae098)
Supplement: evae098_Supplementary_Data [file evae098_supplementary_data.zip › Supplementary_materials.zip!/selected_miRNAs/NoGe_selected_miRNAs/mirdeep_pdf_files/NMRB01001254.1_Notospermus_geniculatus_strain_Ushimado_scaffold1254,_whole_genome_shotgun_sequence_26704.pdf]

Provisional ID	:	NMRB01001254.1_Notospermus_geniculatus_strain_Ushimado_scaffold1254,_whole_genome_shotgun_sequence_26704
Score total	:	1635.3
Score for star read(s)	:	3.9
Score for read counts	:	1627.5
Score for mfe	:	0.9
Score for randfold	:	0
Score for cons. seed	:	3
Total read count	:	3204
Mature read count	:	3197
Loop read count	:	0
Star read count	:	7

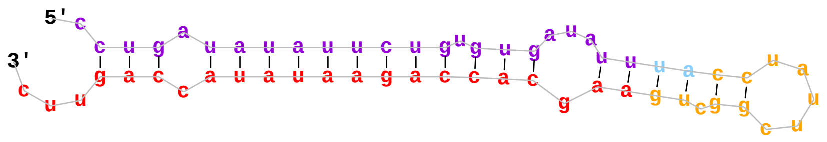

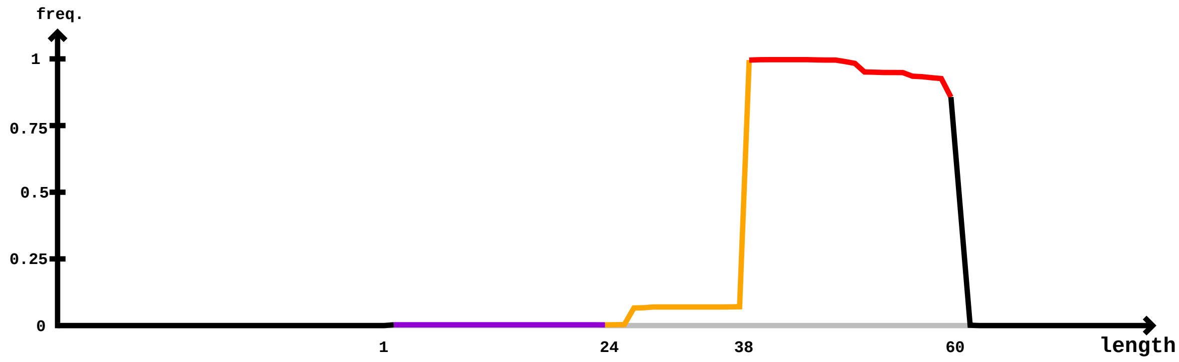

Star Mature

```
5'- gccugccgccguaguagcaugcaugucaugugaccugauauauucugugugauauuuaccuauucggcugaagcaccagaauauaccaguucauuugcuugcaaugaucauc  -3'   obs
    gccugccgccguaguagcaugcaugucaugugaccugauauauucugugugauauuuaccuauucggcugaagcaccagaauauaccaguucauuugcuugcaaugaucauc        exp
    ((((((....))))..)).((((.(.((.((((.(((.(((((((((.(((...((((((.....)).)))).))))))))))))).))).)))).))).)))).........   reads     mm      sample
    .................................ccugauauauucugugCgauau.........................................................   1         1       Fm3
    ........................................................uaccuauucggcugaagcaccagaa...............................   2         0       Fm3
    ..........................................................ccuauucggcugaagcaccaga................................   1         0       Fm3
    ..........................................................ccuauucggcugaagcaccagaau..............................   11        0       Fm3
    ......................................................................aagcaccagaauauacca........................   5         0       Fm3
    ......................................................................aagcaccagaauauaccag.......................   12        0       Fm3
    ......................................................................aagcaccagaauauaccagG......................   1         1       Fm3
    ......................................................................aagcaccagaauauaccagu......................   3         0       Fm3
    ......................................................................aagcaccagGauauaccaguu.....................   2         1       Fm3
    ......................................................................aagcaccagaGuauaccaguu.....................   1         1       Fm3
    ......................................................................aagcaccagaauauaccaguu.....................   41        0       Fm3
    ......................................................................aagcaccagaauauaccUguu.....................   1         1       Fm3
    ......................................................................aagcaccagaaCauaccaguu.....................   1         1       Fm3
    ......................................................................aagcaccagGauauaccaguuc....................   9         1       Fm3
    ......................................................................aagcaccagaauauaccaguuA....................   3         1       Fm3
    ......................................................................aagcaccagaauauaccaguuc....................   39        0       Fm3
    ......................................................................aagcaccagaauauaccaguuca...................   2         0       Fm3
    ......................................................................aagcaccagGauauaccaguuca...................   2         1       Fm3

    .................................ccugauauauucugugugauauu........................................................   1         0       Mm3
    ..........................................................ccuauucggcugaagcacc...................................   2         0       Mm3
    ..........................................................ccuauucggcugaagcaccaga................................   9         0       Mm3
    ..........................................................ccuauucggcugaagcaccagaa...............................   5         0       Mm3
    ..........................................................ccuauucggcugaagcaGcagaau..............................   1         1       Mm3
    ..........................................................cGuauucggcugaagcaccagaau..............................   1         1       Mm3
    ..........................................................ccuauucggcugaagcaccagaau..............................   24        0       Mm3
    ..........................................................ccuauucggcugaagcaccagaaA..............................   1         1       Mm3
    ..........................................................ccuauucggcugaagcaccagaauauac..........................   1         0       Mm3
    ..........................................................ccuauucggcugaagcaccagaauauacc.........................   12        0       Mm3
    ..........................................................ccuauucggcugaagcaccagaauauaccagu......................   1         0       Mm3
    ...........................................................cuauucggcugaagcaccagaauauaccaguuc....................   1         0       Mm3
    .....................................................................Caagcaccagaauauaccaguuc....................   1         1       Mm3
    ......................................................................aagcaccagaauauaccaguu.....................   23        0       Mm3
```

Star Mature

```
gccugccgccguaguagcaugcaugucaugugaccugauauauucugugugauauuuaccuauucggcugaagcaccagaauauaccaguucauuugcuugcaaugaucauc
......................................................................aagcaccagaauauaccaguA.....................	1	1	Mm3
......................................................................aagcaccagaauauaccGguu.....................	1	1	Mm3
......................................................................aagUaccagaauauaccaguuc....................	1	1	Mm3
......................................................................aagcaccagUauauaccaguuc....................	1	1	Mm3
......................................................................aagcaccagGauauaccaguuc....................	14	1	Mm3
......................................................................aagcaccagaauauacAaguuc....................	1	1	Mm3
......................................................................aagcaccagaauauaccaguuA....................	1	1	Mm3
......................................................................aagcaccagaCuauaccaguuc....................	1	1	Mm3
......................................................................aagcaccagaauaAaccaguuc....................	1	1	Mm3
......................................................................aagcaccagaauauaccaguuU....................	1	1	Mm3
......................................................................aagcaccagaauauacGaguuc....................	1	1	Mm3
......................................................................aagcaccagaauauaccaguuc....................	355	0	Mm3
......................................................................aagcaccagaGuauaccaguuc....................	1	1	Mm3
......................................................................aagcaccagaauauaccCguuc....................	1	1	Mm3
......................................................................aagcaccagaauauaccGguuc....................	2	1	Mm3
......................................................................aagcaccagaauauaccUguuc....................	1	1	Mm3
......................................................................aagcaccagaauauaccaguuca...................	7	0	Mm3
......................................................................aagcaccagaauauaccaguucC...................	1	1	Mm3
......................................................................aagcaccagGauauaccaguuca...................	2	1	Mm3
......................................................................aagcaccagaauauaccaguucU...................	18	1	Mm3
......................................................................aagcaccagaauauaccaguucau..................	2	0	Mm3
......................................................................aagcaccagaauauaccaguucUu..................	1	1	Mm3
.......................................................................agcaccagaauauaccaguuc....................	1	0	Mm3
........................................................................gcaccagaauauaccaguuc....................	1	0	Mm3

..........................................................ccuauucggcugaagcaAcagaau..............................	1	1	Fm2
..........................................................ccuauucggcugaagcaccagaau..............................	12	0	Fm2
..........................................................ccuauucggcugaagcaccagaauaAacc.........................	1	1	Fm2
..........................................................ccuauucggcGgaagcaccagaauauacc.........................	1	1	Fm2
..........................................................ccuauucggcugaagcaccagaauauacc.........................	14	0	Fm2
......................................................................aagcaccagaauauaccagu......................	3	0	Fm2
......................................................................aagcaccagaauauaccaguu.....................	22	0	Fm2
......................................................................aagcaccagaauauaccaguuc....................	175	0	Fm2
......................................................................aagcaccagGauauaccaguuc....................	19	1	Fm2
......................................................................aagcaccagaauauaccCguuc....................	1	1	Fm2
......................................................................aagcaccagaauauaccaguuA....................	18	1	Fm2
......................................................................aagcaccagaauauacAaguuc....................	2	1	Fm2
......................................................................aagcaccagaauauaccGguuc....................	1	1	Fm2
......................................................................aagcaccagaauauaccaguucC...................	14	1	Fm2
......................................................................aagcaccagGauauaccaguuca...................	1	1	Fm2
......................................................................aagcaccagaauauaccaguucU...................	17	1	Fm2
......................................................................aagcaccagaauauacAaguuca...................	1	1	Fm2
......................................................................aagcaccagaauauaccaguuca...................	50	0	Fm2
......................................................................aagcaccagaUuauaccaguucau..................	1	1	Fm2

.................................ccugauauauucugugugauauuuaccuauucggcug..........................................	1	0	Fm1
......................................................................aagcaccagaauauaccagAu.....................	1	1	Fm1
......................................................................aagcaccagaauauaccaguu.....................	16	0	Fm1
......................................................................aagcaccagGauauaccaguuc....................	31	1	Fm1
......................................................................aagcaccagaauauaccaguuc....................	88	0	Fm1
......................................................................aagcaccagaauauaccaguuca...................	5	0	Fm1
......................................................................aagcaccagaUuauaccaguuca...................	1	1	Fm1
......................................................................aagcaccagGauauaccaguuca...................	4	1	Fm1

..........................................................ccuauucggcugaagcaccagaa...............................	5	0	Mm1
..........................................................ccuauucggcugaagcaccagaau..............................	5	0	Mm1
..........................................................ccuauucggcugaagcaccagaauUu............................	4	1	Mm1
..........................................................ccuauucggcugaagcaccagaauauacc.........................	3	0	Mm1
..........................................................ccuauucggcugaagcaAcagaauauacc.........................	1	1	Mm1
............................................................uauucggcugaagcaccagaauauaccaguuc....................	1	0	Mm1
......................................................................aagcaccagaauauaccagG......................	1	1	Mm1
......................................................................aagcaccagaauauaccagu......................	1	0	Mm1
......................................................................aagcaccagaauauaccGguu.....................	1	1	Mm1
......................................................................aagcaccagaauauaccaguu.....................	46	0	Mm1
......................................................................aagcaccagaUuauaccaguuc....................	2	1	Mm1
......................................................................aagcaccagaauauaccGguuc....................	1	1	Mm1
......................................................................aagcaccagaCuauaccaguuc....................	1	1	Mm1
......................................................................aagcaccagaauauaccaguuc....................	83	0	Mm1
......................................................................aagcaccagGauauaccaguuc....................	13	1	Mm1
......................................................................aagcaccagaauauaccaguucU...................	3	1	Mm1
```

```
                                Star                                Mature
gccugccgccguaguagcaugcaugucaugugaccugauauauucugugugauauuuaccuauucggcugaagcaccagaauauaccaguucauuugcuugcaaugaucauc
......................................................................aagcaccagGauauaccaguuca...................   1      1    Mm1
......................................................................aagcaccagaauauaccaguuca...................   6      0    Mm1

.................................ccugauauauucugugugauauu........................................................   4      0    Mm2
.................................ccugauauauucugugugauauuu.......................................................   1      0    Mm2
.................................ccugauauauucugugugauauuuaccuauucggcug..........................................   2      0    Mm2
........................................................uaccuauucggcugaagcacca..................................   2      0    Mm2
........................................................uaccuauucggcugaagcaccagU................................   1      1    Mm2
........................................................uaccuauucggcugaagcaccaga................................   4      0    Mm2
..........................................................ccuauucggcugaagcacc...................................   1      0    Mm2
..........................................................ccuauucggcugaagcaccaga................................   5      0    Mm2
..........................................................ccuauucggcugaagcUccagaa...............................   1      1    Mm2
..........................................................ccuauucggcugaagcaccagaa...............................   10     0    Mm2
..........................................................ccuauucggcugaagcaGcagaau..............................   1      1    Mm2
..........................................................ccuauucggcugaagcaccagaau..............................   54     0    Mm2
..........................................................ccuauucggcugaagcaccagaauU.............................   1      1    Mm2
..........................................................ccuauucggcugaagcaccagaaua.............................   2      0    Mm2
..........................................................ccuauucggcugaagcaccagaauauacc.........................   15     0    Mm2
..........................................................ccuauucggcugaagcaccagaauauaccaguuc....................   7      0    Mm2
..........................................................ccuauucggcugaagcaccagaauauaccGguuc....................   1      1    Mm2
..........................................................ccuauucggcugaagcaAcagaauauaccaguuc....................   1      1    Mm2
...........................................................cuauucggcugaagcaccagaauauaccaguuc....................   2      0    Mm2
............................................................uauucggcugaagcaccagaauauaccag.......................   1      0    Mm2
............................................................uauucggcugaagcaccagaauauaccaguuc....................   8      0    Mm2
....................................................................ugaagcaccagaauauaccaguuc....................   1      0    Mm2
.....................................................................Caagcaccagaauauaccaguuc....................   1      1    Mm2
......................................................................aagcaccagaauauacca........................   1      0    Mm2
......................................................................aagcaccagaauauaccagu......................   1      0    Mm2
......................................................................aagcaccagaauauaccaguu.....................   80     0    Mm2
......................................................................aagcaccagGauauaccaguu.....................   1      1    Mm2
......................................................................aagcaccagaGuauaccaguu.....................   1      1    Mm2
......................................................................aagcaccagaUuauaccaguu.....................   1      1    Mm2
......................................................................aagcaccagaauauacUaguuc....................   8      1    Mm2
......................................................................Nagcaccagaauauaccaguuc....................   1      1    Mm2
......................................................................aagcaccagaauauaccCguuc....................   2      1    Mm2
......................................................................aagcaccagGauauaccaguuc....................   77     1    Mm2
......................................................................aagcaNcagaauauaccaguuc....................   1      1    Mm2
......................................................................aagcaccagaauauaccGguuc....................   3      1    Mm2
......................................................................aagcaccagaauauacAaguuc....................   5      1    Mm2
......................................................................aagcaccagaauauacGaguuc....................   1      1    Mm2
......................................................................aagcaccagaauauaccaguuU....................   4      1    Mm2
......................................................................aaUcaccagaauauaccaguuc....................   2      1    Mm2
......................................................................Cagcaccagaauauaccaguuc....................   1      1    Mm2
......................................................................aagcaccagaaCauaccaguuc....................   1      1    Mm2
......................................................................aagcaUcagaauauaccaguuc....................   1      1    Mm2
......................................................................aagcaccagaGuauaccaguuc....................   7      1    Mm2
......................................................................Uagcaccagaauauaccaguuc....................   1      1    Mm2
......................................................................aagcaccagCauauaccaguuc....................   1      1    Mm2
......................................................................aagcaccagaUuauaccaguuc....................   11     1    Mm2
......................................................................aagcacUagaauauaccaguuc....................   1      1    Mm2
......................................................................aagcaccagUauauaccaguuc....................   2      1    Mm2
......................................................................aagcaccagaauauaccUguuc....................   5      1    Mm2
......................................................................aagcaccagaauauaccaguuc....................   1695   0    Mm2
......................................................................aagcaccagaauauaccaguuG....................   1      1    Mm2
......................................................................aagcaccagaauaCaccaguuc....................   1      1    Mm2
......................................................................aagcaccUgaauauaccaguuc....................   1      1    Mm2
......................................................................aagcaccagaauauacUaguuca...................   1      1    Mm2
......................................................................aagcaccagaauauaccaguucU...................   37     1    Mm2
......................................................................aagcaccagGauauaccaguuca...................   5      1    Mm2
......................................................................aagcaccagaauauaccaguuca...................   37     0    Mm2
.......................................................................agcaccagaauauaccaguuc....................   4      0    Mm2
```
